# Epidemiology and Outcomes of Pediatric Fever in a Rural District of Southern Mozambique: 17 Years of Morbidity Surveillance

**DOI:** 10.1093/ofid/ofaf724

**Published:** 2025-11-28

**Authors:** David Torres-Fernandez, Jessica Dalsuco, Cristina Garcia-Mauriño, Núria Balanza, Marta Valente, Sara Ajanovic, Rosauro Varo, Jaime Fanjul, Justina Bramugy, Antonio Sitoe, Llorenç Quintó, Tacilta Nhampossa, Edith Taylor, Fio Vialard, Arsenio Nhacolo, Bàrbara Baro, Anelsio Cossa, Zumilda Boca, Sergio Massora, Andrea Alemany, Inácio Mandomando, Pere Millat-Martinez, Pedro Aide, Quique Bassat

**Affiliations:** Centro Investigação em Saúde de Manhiça (CISM), Maputo, Mozambique; ISGlobal, Barcelona, Spain; Facultat de Medicina i Ciencies de la Salut, Universitat de Barcelona (UB), Barcelona, Spain; Centro Investigação em Saúde de Manhiça (CISM), Maputo, Mozambique; ISGlobal, Barcelona, Spain; ISGlobal, Barcelona, Spain; Facultat de Medicina i Ciencies de la Salut, Universitat de Barcelona (UB), Barcelona, Spain; Centro Investigação em Saúde de Manhiça (CISM), Maputo, Mozambique; ISGlobal, Barcelona, Spain; Centro Investigação em Saúde de Manhiça (CISM), Maputo, Mozambique; ISGlobal, Barcelona, Spain; Facultat de Medicina i Ciencies de la Salut, Universitat de Barcelona (UB), Barcelona, Spain; ISGlobal, Barcelona, Spain; Facultat de Medicina i Ciencies de la Salut, Universitat de Barcelona (UB), Barcelona, Spain; ISGlobal, Barcelona, Spain; Centro Investigação em Saúde de Manhiça (CISM), Maputo, Mozambique; Centro Investigação em Saúde de Manhiça (CISM), Maputo, Mozambique; Centro Investigação em Saúde de Manhiça (CISM), Maputo, Mozambique; ISGlobal, Barcelona, Spain; Centro Investigação em Saúde de Manhiça (CISM), Maputo, Mozambique; Instituto Nacional de Saúde (INS), Maputo, Mozambique; ISGlobal, Barcelona, Spain; Facultat de Medicina i Ciencies de la Salut, Universitat de Barcelona (UB), Barcelona, Spain; ISGlobal, Barcelona, Spain; Facultat de Medicina i Ciencies de la Salut, Universitat de Barcelona (UB), Barcelona, Spain; Centro Investigação em Saúde de Manhiça (CISM), Maputo, Mozambique; ISGlobal, Barcelona, Spain; Centro Investigação em Saúde de Manhiça (CISM), Maputo, Mozambique; Centro Investigação em Saúde de Manhiça (CISM), Maputo, Mozambique; Centro Investigação em Saúde de Manhiça (CISM), Maputo, Mozambique; ISGlobal, Barcelona, Spain; Centro Investigação em Saúde de Manhiça (CISM), Maputo, Mozambique; Instituto Nacional de Saúde (INS), Maputo, Mozambique; ISGlobal, Barcelona, Spain; Centro Investigação em Saúde de Manhiça (CISM), Maputo, Mozambique; Centro Investigação em Saúde de Manhiça (CISM), Maputo, Mozambique; ISGlobal, Barcelona, Spain; Facultat de Medicina i Ciencies de la Salut, Universitat de Barcelona (UB), Barcelona, Spain; ICREA, Pg. Lluis Companys 23, Barcelona, Spain; Pediatrics Department, Hospital Sant Joan de Deu, Universitat de Barcelona, Esplugues, Barcelona, Spain; CIBER de Epidemiología y Salud Pública, Instituto de Salud Carlos III, Madrid, Spain; Institut Clinic de Medicina i Dermatologia, Hospital Clinic de Barcelona, Barcelona, Spain

**Keywords:** fever, infectious disease, low- and middle-income countries, mortality, pediatrics

## Abstract

**Background:**

Pediatric febrile illnesses remain a leading cause of health care visits, morbidity, and mortality in low-resource settings. Their etiological diagnosis and outcome evaluation are challenging as available tools are limited. Herein, we describe the epidemiology, trends, and clinical outcomes of febrile pediatric outpatient clinic visits and inpatients during a 17-year-long period in Southern Mozambique.

**Methods:**

We retrospectively analyzed surveillance morbidity and demographic data from children <15 years old presenting with fever (≥37.5°C) at Manhiça District Hospital and 5 peripheral health posts from 2004 to 2020. We characterized diagnoses and clinical signs, stratified by outpatient clinic visits and hospitalizations, and calculated 7-day mortality odds, case fatality ratios (CFRs), and minimum community-based incidence rates.

**Results:**

A total of 664 223 outpatient visits and 23 166 hospitalizations were included. The median age (interquartile range) was 47.1 (20.0–92.3) months for outpatients and 21.2 (10.1–41.5) months for inpatients. The most frequent first encounter diagnoses included malaria (33.5%), upper (27.8%) and lower (10.1%) respiratory tract infections, and acute gastrointestinal infection (6.0%), whose frequencies showed a marked annual decline from 2004 to 2020, particularly among inpatients. All-cause 7-day mortality was 0.1% and 2.2% among outpatients and inpatients, respectively. Sepsis and meningitis were less common but presented the highest CFRs (9%–16%). Malnutrition and HIV infection were major contributors to inpatient mortality. Seizures, edema, dehydration, and reduced consciousness were strong predictors of death.

**Conclusions:**

Malaria, respiratory tract, and acute gastrointestinal infections represented the predominant causes of fever and mortality, with decreasing trends over time. This analysis underscores the value of epidemiological surveillance and the need for improved early diagnosis and clinical management tools for febrile children.

Fever is one of the most frequent symptoms in children, leading to seeking health care and triggering hospitalization, with >1 billion annual episodes globally [1]. Fever is particularly common in Sub-Saharan Africa, where each child under 5 years old (U5) experiences ∼6 episodes per year [2, 3]. Fever is the main manifestation of most infectious diseases, which are responsible for over two-thirds of the 4.8 million annual child deaths. These deaths are disproportionately concentrated in low- and middle-income countries (LMICs), resulting in unacceptable negative health and economic impacts [4].

While most infections are uncomplicated, a small proportion (<1%) can be life-threatening, requiring early recognition and prompt management to prevent mortality [1]. In high-income countries, immediate management decisions are feasible due to increased resources, including comprehensive etiological research, highly specialized medical training, advanced diagnostic tests, and continuous availability of essential medication. However, in LMICs, where these resources are scarce, etiological diagnosis and outcome evaluation of children with fever are suboptimal, jeopardizing appropriate case management [5]. Triage errors can result in misdiagnosis and, more importantly, failure to recognize disease severity [6, 7]. Conversely, unnecessary admissions and treatment of self-limiting infections can lead to overburdened health systems, increased rates of nosocomial infections, inappropriate antimicrobial use, and, consequently, antimicrobial resistance [2, 8–12].

Malaria has played a dominant role in the assessment of febrile episodes in malaria-endemic regions. The 2010 World Health Organization (WHO) recommendation that all patients with suspected malaria be tested before treatment marked a turning point in the global approach to fever evaluation [1]. Previous studies have focused on distinguishing malaria from nonmalarial fevers [13, 14], identifying diagnostic errors due to overlapping conditions such as severe malaria and pneumonia [15], and assessing increased mortality risk associated with comorbidities like malnutrition or HIV infection [16, 17]. Few studies have investigated the etiological causes of fever in LMICs [12, 17–21]. These have often been limited by short study periods (hindering longitudinal trend assessment), restricted populations, focus on single infections, and confinement to either outpatients or inpatients and thus have lacked a comprehensive epidemiological understanding. In children from LMICs, frequently reported causes of fever include upper (URTI) and lower (LRTI) respiratory tract infections, malaria, acute gastrointestinal infection (AGI), and invasive bacterial infection, though the majority of fevers may be caused by relatively benign viruses [14]. In newborns, a particularly vulnerable group, bacteremia is a greater cause of nonmalarial febrile illness [14]. Despite extensive testing, the etiology of fever often remains unknown, and thorough reports linking clinical diagnoses to mortality remain rare in LMICs [22]. Understanding the distribution of clinical syndromes, diagnosis, and outcomes in both outpatient and hospitalized children is essential, particularly because many of these conditions are preventable.

In this study, we aimed to describe the epidemiology, trends, and clinical outcomes of febrile pediatric outpatient clinic visits and inpatients from a district hospital and 5 peripheral health centers in Southern Mozambique, including the associated 7-day mortality odds for different diagnoses and clinical signs, using 17 years of morbidity and demographic surveillance data.

## METHODS

### Study Site

The district of Manhiça is located in Southern Mozambique, ∼90 km north of Maputo, the capital city, and has been thoroughly described elsewhere [23–25]. In short, it is a resource-constrained, semirural area covering 2380 km^2^. In 2022, the total population was ∼209 000, predominantly young individuals (18% U5). Over the years, the U5 mortality rate in the district has declined, from 58.5 per 1000 live births in 2014 to 31.3 per 1000 in 2020 [25]. *Plasmodium falciparum* malaria transmission is perennial in the area, with peaks during the rainy season (November to April), and its incidence has markedly decreased over time [26].

Health care in the district of Manhiça is provided by Manhiça District Hospital (MDH), Xinavane Rural Hospital, and several outpatient clinics. MDH serves both as a primary health care center for the nearby population and as a referral center for the broader district. The peripheral health posts have very basic medical equipment and limited supplies and do not provide inpatient care. All clinical management and treatment are free of charge, except for a standard subsidized fee for outpatient medications. Access to specialized health care services is limited [24, 25].

### Demographic and Morbidity Surveillance System

The Manhiça Health Research Center (Centro de Investigação em Saúde de Manhiça [CISM]) has run a health and demographic surveillance system since 1996, comprising both demographic surveillance (DSS) for residents in Manhiça district and morbidity surveillance (MSS) at health care facilities [23]. The catchment area of DSS has progressively expanded and has covered the entire district since 2014. Each resident is assigned a unique permanent identification number (“perm_ID”). The DSS collects sociodemographic data and other major life events and is updated annually [25]. The MSS involves 7 health care facilities in Manhiça district. Each time a patient aged <15 years attends a health care facility, a standardized paper-based form is completed with information about the health event. These forms include demographic and clinical information, such as symptoms, diagnoses coded in the International Classification of Diseases, 10th Revision (ICD-10), format [27], and outcomes. MSS forms are linked to the DSS through the perm_ID [25].

### Study Design

This study is a retrospective analysis of morbidity and demographic surveillance data from the Manhiça MSS and DSS aimed at describing the epidemiology, trends, and outcomes of febrile illnesses in pediatric outpatient visits and admitted patients. We included children aged <15 years with registered fever (≥37.5°C) or self-reported high body temperature (“referred” fever) and residents in Manhiça District with an identification number (perm_ID) presenting to the outpatient clinic at MDH or 5 peripheral health posts (Maragra, Malavele, Palmeira, Taninga, and Ilha Josina Machel), as well as those admitted as inpatients at MDH, from January 1, 2004, to December 31, 2020. Fever was not a requirement for inclusion for patients <2 months of age due to the nonspecificity of symptoms and vulnerability of this age group. We only included 5 out of 7 health posts and excluded patients from Xinavane Rural Hospital for data integrity and traceability reasons. Taninga Clinic was incorporated into the MSS in 2005, while Palmeira and Malavele were incorporated in 2009. A clinical episode was defined as all visits of a patient occurring within 7 days of an index visit (ie, a patient's attendance to the outpatient clinic). If a clinical episode included >1 visit, only the last visit was considered for analysis to avoid duplication and to reflect the most accurate diagnosis and later-stage presentation. Notably, all inpatients were initially evaluated in an outpatient clinic visit. For outpatient-related results, we used data from outpatient visit forms, and for inpatients, we used data from inpatient forms. We classified patients into 4 age subgroups: neonate (0 to 27 days), infant (28 days to <1 year), early childhood (≥1 to <5 years), and late childhood (≥5 to <15 years).

The outpatient clinic at MDH or at peripheral centers included a clinical examination, malaria testing, and, occasionally, basic laboratory testing (hematocrit or full blood count), similar to an emergency room in a resource-limited setting. At MDH, a short-stay unit was available to monitor patients during the day and provide more advanced treatments, such as intravenous medications or nebulizers, before hospitalization. Inpatients were admitted to the pediatric ward, available only at MDH. During admission, additional diagnostic investigations were available, including chest x-rays, blood biochemistry, blood cultures, and mycobacterial identification at CISM's ISO-certified laboratory. Severely ill patients at MDH needing advanced care (eg, intensive care or specialized evaluation) could be referred to Maputo Central Hospital in the capital city. Up to 4 diagnoses were coded by clinical judgment using the ICD-10 format in the MSS forms. Multiple specifications of similar ICD-10 diagnostic codes were grouped for analysis per the authors’ criteria (Supplementary Data 1). Severe underweight was defined as weight-for-age Z-score <−3, based on Centers for Disease Control and Prevention growth charts [28]. Malaria diagnosis required additional confirmatory diagnostic testing (Supplementary Data 1).

### Data Analysis

We reported descriptive data using counts, percentages, medians, and interquartile ranges (IQRs). We included sociodemographic variables, clinical history, physical examination findings, and diagnoses recorded in the MSS forms. We stratified results for outpatient visits and inpatients and described diagnostic information according to age group. A complete case analysis approach was used, and missing data were reported.

We calculated annual mortality proportions for outpatient visits and inpatients, case fatality ratios (CFRs), and minimum community-based incidence rates (MCBIRs) for diagnoses stratified by age group. Notably, MCBIRs included only children residing in the original CISM DSS catchment area (pre-expansion) to ensure year-to-year comparability (Supplementary Data 1) [24].

We evaluated the association between clinical signs and diagnosis with 7-day mortality by fitting separated multivariable logistic regression models, stratified by outpatient and inpatient status. The outcome was a binary variable indicating death within 7 days of the outpatient visit. We calculated adjusted odds ratios (aORs), 95% CIs, and *P* values. All models were adjusted for age group, sex, and malaria season as possible confounders. The reference group was male patients in the late childhood and dry season who were not presenting with the listed diagnoses. Separation was handled with the exclusion of clinically irrelevant categories or those with sparse data. All statistical analyses were performed using R, version 4.4 [29], except for MCBIRs, which were calculated using STATA, version 15 (StataCorp LLC). This study had ethical approval from corresponding institutions in Mozambique (Supplementary Data 1).

## RESULTS

### Population Description, Clinical Management, and Outcomes

A total of 664 223 outpatient visits (representing 97 155 unique patients) and 23 166 hospitalizations (16 689 unique patients) recorded between January 1, 2004, and December 31, 2020, were included in the analysis. The median age (IQR) was 47.1 (20.0–92.3) months for outpatients and 21.2 (10.1–41.5) months for inpatients. The most frequent age group was early childhood in both outpatients (290 892 [44.2%]) and inpatients (12 741 [55.2%]), followed by late childhood (273 919 [41.7%]) in outpatients and infants (6178 [26.8%]) in inpatients. Approximately half of the total patients (49.5% outpatients, 44.5% inpatients) were female. Of all outpatient visits, 287 354 (43.3%) occurred at MDH. At triage, most patients were discharged home (619 537 [93.4%]), while 40 746 (6.1%) were admitted to the Short Stay Unit. Of those, 24 964 (61.2%) were subsequently hospitalized, representing 3.8% of all outpatient visits. Following hospitalization, most patients were discharged home alive (20 842 [90.7%]); however, 646 (2.8%) died, 742 (3.2%) absconded, and 754 (3.3%) were transferred to the capital city (Table 1). Mortality within 7 days of an outpatient visit was 0.1% for outpatients (933/664 223) and 2.2% for inpatients (515/23 166). Within 30 days, it was 0.2% for outpatients (1572/664 223) and 3.8% for inpatients (887/23 166).

**Table 1.** Population Description and Management for Visits to Outpatient Clinic and Inpatients

	Outpatients	Inpatients
	n = 664 223, No. (%)	n = 23 166, No. (%)
Age, median (IQR), mo	47.1 (20.0–92.3)	21.2 (10.1–41.5)
Missing	6805	71
Age groups	…	…
Late childhood (≥5 to <15 y)	273 919 (41.7)	3446 (14.9)
Early childhood (≥1 to <5 y)	290 892 (44.2)	12 741 (55.2)
Infant (28 d to <1 y)	90 578 (13.8)	6178 (26.8)
Neonate (0 to 27 d)	2029 (0.3)	730 (3.2)
Missing	6805	71
Sex, female	328 629 (49.5)	10 395 (44.9)
Missing	63	0
Season, rainy	406 402 (61.2)	14 798 (63.9)
Place of visit		
Manhiça District Hospital (MDH)	287 354 (43.3)	…
Maragra Health Center	122 623 (18.5)	…
Ilha Josina Health Center	82 493 (12.4)	…
Taninga Health Center	44 248 (6.7)	…
Palmeira Health Center	82 602 (12.4)	…
Malavele Health Center	44 903 (6.8)	…
Management at triage (MDH and peripheral centers)	…	…
Home	619 537 (93.4)	…
Admitted to short stay unit at MDH	40 746 (6.1)	…
Transferred to MDH	1929 (0.3)	…
Absconded	1444 (0.2)	…
Missing	567	…
Management at short stay unit (only MDH)	n = 40 812	…
Home	15 114 (37.0)	…
Admitted to pediatric ward at MDH	24 964 (61.2)	…
Transferred to Maputo Central Hospital	508 (1.2)	…
Death	50 (0.1)	…
Absconded	176 (0.4)	…
Management at pediatric ward (only MDH)	…	…
Discharged home alive	…	20 842 (90.7)
Death	…	646 (2.8)
Absconded	…	742 (3.2)
Transferred	…	754 (3.3)
Missing	…	182
Length of hospital stay, median (IQR), d	…	3.0 (2.0–5.0)
Missing	…	66

Abbreviations: IQR, interquartile range; MDH, Manhiça District Hospital.

The total number of outpatient visits (per 1000 children aged <15 years) varied throughout the study period, with ∼9000 visits per year from 2004 to 2010, peaking between 2011 and 2014, and then declining to 2932 visits in 2020. The number of admitted patients (per 1000 children aged <15 years) steadily decreased from 729 in 2004 to 435 in 2020. Mortality among outpatients decreased from 0.29% in 2004 to 0.1% in 2020, while inpatient mortality varied between 1.2% and 2.9% (Supplementary Data 2).

### Clinical Signs

The median number of previous days with referred fever (IQR) was 1.0 (1.0–2.0) for outpatients and 1.0 (1.0–3.0) for inpatients. Severe underweight was present in 5.1% of outpatients and 13.6% of inpatients. The most commonly observed clinical signs were related to respiratory illnesses. Cough was present in 55.5% of outpatients and 63.2% of inpatients, but severity signs were more common among inpatients, including tachypnea (44.4%) and chest indrawing (21.3%). Diarrhea and vomiting were recorded in 6.9% and 8.4% of outpatients, respectively, and in 20.4% and 23.7% of inpatients. Moderate to severe dehydration was less frequent but more common among inpatients (8.4% vs 0.3% of outpatients). Regarding neurological signs, seizures and reduced levels of consciousness were observed in 0.5%–0.6% of outpatients and 13.4%–13.6% of inpatients, respectively (Table 2).

**Table 2.** Clinical Signs in Outpatient Clinic Visits and Inpatients

	Outpatients	Inpatients
	n = 664 223, No. (%)	n = 23 166, No. (%)
Measured temperature, median (IQR), °C	37.1 (36.5–38.1)	38.0 (37.0–39.0)
Missing	333	70
Measured fever (axillary temperature ≥37.5°C)	272 164 (41.0)	14 771 (64.0)
Missing	333	70
Days with fever, median (IQR)	1.0 (1.0–2.0)	1.0 (1.0–3.0)
Missing	1145	635
Cough	367 937 (55.5)	14 628 (63.2)
Missing	1320	6
Days with cough, median (IQR)	2.0 (1.0–3.0)	2.0 (1.0–3.0)
Missing	296 773	8544
History of breathing difficulties	8237 (1.2)	5231 (22.7)
Missing	1221	110
Tachypnea	75 729 (11.6)	10 218 (44.4)
Missing	12 590	166
Chest indrawing	6291 (0.9)	4923 (21.3)
Missing	1020	20
Nasal flaring	5040 (0.8)	3675 (15.9)
Missing	1027	4
Crackles	23 363 (3.5)	4996 (21.6)
Missing	1132	16
Wheezing or rhonchus	49 404 (7.5)	3306 (14.3)
Missing	1156	4
Diarrhea	45 788 (6.9)	4726 (20.4)
Missing	1287	6
Days with diarrhea, median (IQR)	1.0 (1.0–1.0)	1.0 (1.0–2.0)
Missing	618 472	18 461
Stool characteristics	…	…
Watery	43 906 (96.5)	3953 (86.3)
Bloody	1602 (3.5)	627 (13.7)
Missing	618 715	18 586
Vomits	55 576 (8.4)	5479 (23.7)
Missing	1223	9
Days with vomits, median (IQR)	1.0 (1.0–1.0)	1.0 (1.0–1.0)
Missing	608 743	17 711
Prolonged skin fold recovery	1938 (0.3)	1935 (8.4)
Missing	1015	6
Dehydration	…	…
No	656 106 (98.9)	19 306 (83.4)
Mild	5058 (0.8)	1917 (8.3)
Moderate	1606 (0.2)	1427 (6.2)
Severe	444 (0.1)	503 (2.2)
Missing	1009	13
Seizures	3779 (0.6)	3153 (13.6)
Missing	1315	28
No. of seizures in the last 24 h, median (IQR)	1.0 (1.0–2.0)	1.0 (1.0–2.0)
Missing	660 590	20 045
Fontanelle	…	…
Normal	…	9224 (90.0)
Sunken	…	867 (8.5)
Tense	…	158 (1.5)
Missing	…	12 917
Neck stiffness	155 (0.0)	180 (0.8)
Missing	1022	10
Lethargy (outpatients)/prostration (inpatients)	3359 (0.5)	3093 (13.4)
Missing	1031	3
Blantyre Coma Scale	…	…
0	…	50 (0.2)
1	…	90 (0.4)
2	…	324 (1.4)
3	…	329 (1.4)
4	…	674 (2.9)
5	…	21 664 (93.7)
Missing	…	35
Stopped drinking or breastfeeding	…	1985 (8.6)
Missing	…	140
Stopped eating	…	3084 (14.9)
Missing	…	2509
Pallor	18 203 (2.7)	4297 (18.5)
Missing	1106	0
Jaundice	657 (0.1)	316 (1.4)
Missing	1018	0
Edema	4590 (0.7)	1407 (6.1)
Missing	1019	2
Localization of edema	…	…
Face	…	246 (17.8)
Legs	…	742 (53.8)
Generalized	…	263 (19.1)
Other	…	129 (9.3)
Missing	…	21 786
Weight-for-age Z-score, median (IQR)	−0.9 (−1.7, −0.2)	−1.2 (−2.3, −0.3)
Severe underweight (<3 SD)	33 359 (5.1)	2917 (13.6)
Missing	14 415	1650

Abbreviation: IQR, interquartile range.

The clinical signs with the highest 7-day mortality odds in outpatients were presence of seizures (aOR, 5.95; 95% CI, 4.47–7.83), edema (aOR, 5.84; 95% CI, 4.21–7.94), lethargy (4.52; 95% CI, 3.53–5.77), moderate/severe dehydration (aOR, 4.06; 95% CI, 2.64–6.11), difficulty breathing (aOR, 3.67; 95% CI, 2.74–4.88), and severe underweight (aOR, 3.30; 95% CI, 2.73–3.97). Among inpatients, these included presence of prostration (aOR, 4.15; 95% CI, 3.27–5.25), edema (aOR, 3.15; 95% CI, 2.15–4.51), difficulty breathing (aOR, 2.90; 95% CI, 2.07–4.04), and severe underweight (aOR, 2.62; 95% CI, 2.07–3.31). Pallor and anemia were also associated with increased odds of mortality, particularly in outpatients. Additionally, among outpatients, neonates and infants had higher mortality risk compared with older age groups (Supplementary Data 3).

### Clinical Diagnoses

**Figure 1.**
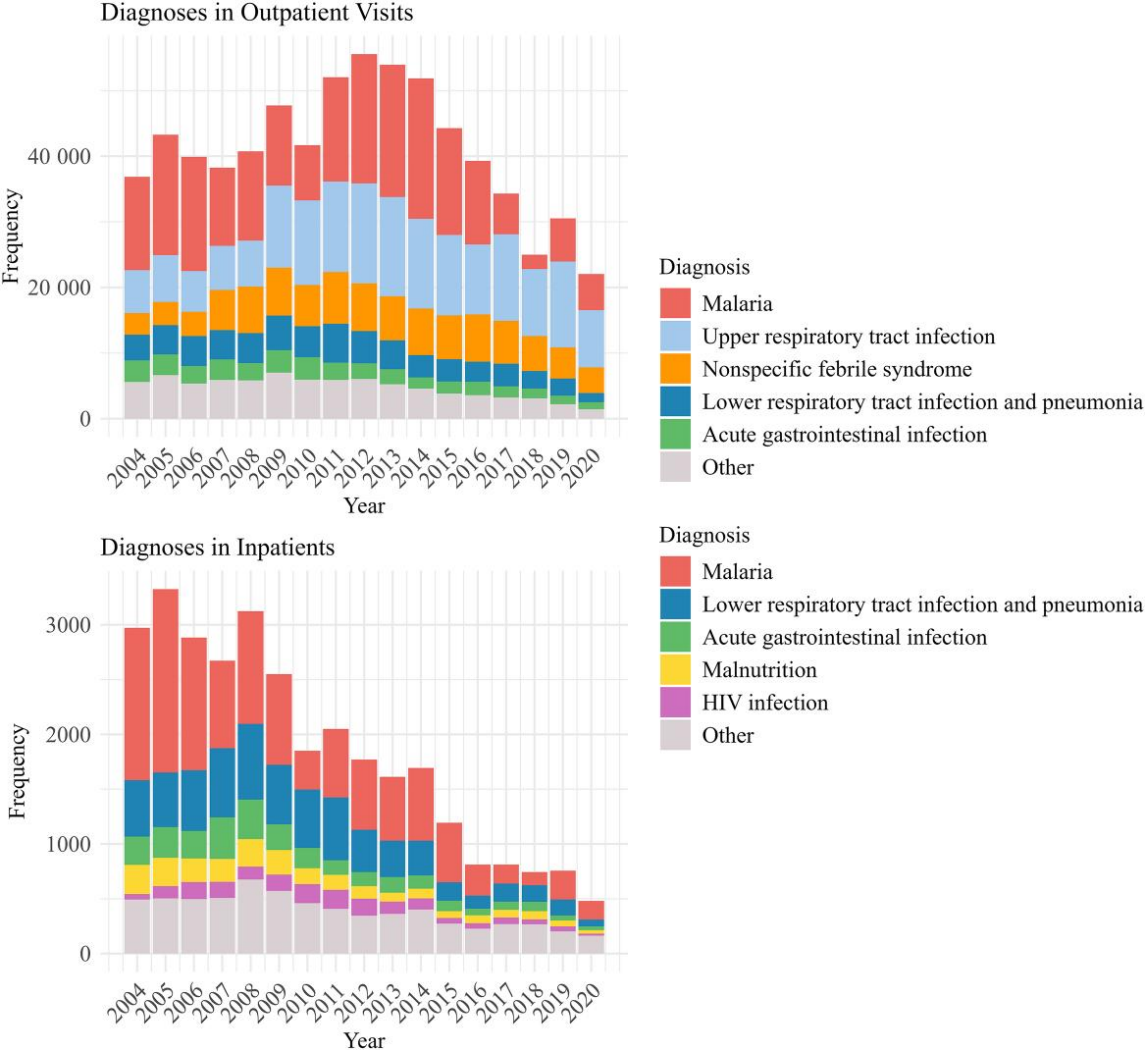
Most common diagnoses per year in outpatient clinic visits and inpatients. Y-axis (frequency) plots the absolute number of records. Anemia diagnosis was excluded from graph.

The total number of diagnoses varied over time, with the overall number of visits and admissions showing a marked annual decline in cases of malaria, LRTI, and AGI, particularly among inpatients (Figure 1). The most frequent diagnoses among outpatients were malaria (33.5%), URTI (27.8%), nonspecific febrile syndrome/unspecified fever (15.2%), LRTI (10.1%), and AGI (6.0%). Among inpatients, the most common diagnoses were malaria (49.0%), LRTI (27.5%), AGI (12.4%), malnutrition (10.2%), HIV infection (7.4%), and seizures with fever (6.7%) (Table 3). URTIs occurred consistently across all outpatient age groups. LRTIs and AGIs were more common among infants and children aged 1 to <5 years in both outpatient and inpatient settings. In contrast, malaria occurred more frequently in older children. In neonates, a high frequency of unspecified fever was observed, accounting for 22.4% of outpatients and 16.6% of inpatients. Overlap of multiple diagnoses was common (Supplementary Data 4), most notably the concurrence of HIV infection and malnutrition with LRTI or *Pneumocystis jirovecii* infection, as well as the overlap of malaria with LRTI, anemia, and seizures with fever.

**Table 3.** Proportion of Diagnoses for Each Age Group, Stratified by Outpatient Clinic Visits and Inpatients

	Outpatients					Inpatients				
	Total Outpatients, No. (%)	Neonate, No. (%)	Infant, No. (%)	Early Child, No. (%)	Late Child, No. (%)	Total Inpatients, No. (%)	Neonate, No. (%)	Infant, No. (%)	Early Child, No. (%)	Late Child, No. (%)
	n = 664 223	n = 2029	n = 90 578	n = 290 892	n = 273 919	n = 23 166	n = 730	n = 6178	n = 12 741	n = 3446
Sepsis	81 (0.0)	31 (1.5)	18 (0.0)	21 (0.0)	10 (0.0)	483 (2.1)	47 (6.4)	174 (2.8)	224 (1.8)	35 (1.0)
Nonspecific febrile syndrome	100 707 (15.2)	455 (22.4)	10 844 (12.0)	37 238 (12.8)	51 070 (18.6)	548 (2.4)	121 (16.6)	128 (2.1)	217 (1.7)	80 (2.3)
Tuberculosis	228 (0.0)	0 (0.0)	32 (0.0)	58 (0.0)	137 (0.1)	304 (1.3)	0 (0.0)	66 (1.1)	144 (1.1)	94 (2.7)
*Pneumocystis*	170 (0.0)	0 (0.0)	41 (0.0)	85 (0.0)	41 (0.0)	77 (0.3)	1 (0.1)	43 (0.7)	28 (0.2)	5 (0.1)
URTI	184 926 (27.8)	547 (27.0)	29 079 (32.1)	83 658 (28.8)	69 530 (25.4)	747 (3.2)	23 (3.2)	211 (3.4)	446 (3.5)	63 (1.8)
LRTI and pneumonia	67 191 (10.1)	243 (12.0)	20 438 (22.6)	35 696 (12.3)	10 185 (3.7)	6379 (27.5)	95 (13.0)	2585 (41.8)	3101 (24.3)	586 (17.0)
Noninfectious respiratory condition	3527 (0.5)	4 (0.2)	577 (0.6)	1934 (0.7)	958 (0.3)	110 (0.5)	2 (0.3)	22 (0.4)	65 (0.5)	21 (0.6)
Acute gastrointestinal infection	40 173 (6.0)	102 (5.0)	14 018 (15.5)	20 899 (7.2)	4736 (1.7)	2862 (12.4)	33 (4.5)	1406 (22.8)	1291 (10.1)	123 (3.6)
Parasitic gastrointestinal infection	15 943 (2.4)	4 (0.2)	394 (0.4)	6890 (2.4)	8558 (3.1)	128 (0.6)	0 (0.0)	19 (0.3)	88 (0.7)	20 (0.6)
Malaria	222 764 (33.5)	131 (6.5)	14 130 (15.6)	92 224 (31.7)	114 274 (41.7)	11 349 (49.0)	50 (6.8)	2186 (35.4)	7143 (56.1)	1935 (56.2)
Meningoencephalitis	239 (0.0)	10 (0.5)	77 (0.1)	77 (0.0)	75 (0.0)	254 (1.1)	21 (2.9)	81 (1.3)	75 (0.6)	77 (2.2)
Seizures with fever	2682 (0.4)	22 (1.1)	237 (0.3)	1847 (0.6)	563 (0.2)	1544 (6.7)	21 (2.9)	163 (2.6)	1108 (8.7)	249 (7.2)
Ear infections	15 827 (2.4)	19 (0.9)	3363 (3.7)	7438 (2.6)	4868 (1.8)	383 (1.7)	2 (0.3)	124 (2.0)	222 (1.7)	34 (1.0)
Ocular infections	17 102 (2.6)	111 (5.5)	4325 (4.8)	8561 (2.9)	3932 (1.4)	126 (0.5)	30 (4.1)	49 (0.8)	40 (0.3)	7 (0.2)
HIV infection	1353 (0.2)	10 (0.5)	250 (0.3)	672 (0.2)	413 (0.2)	1723 (7.4)	9 (1.2)	361 (5.8)	946 (7.4)	402 (11.7)
Malnutrition	3635 (0.5)	23 (1.1)	907 (1.0)	2539 (0.9)	141 (0.1)	2362 (10.2)	8 (1.1)	629 (10.2)	1615 (12.7)	103 (3.0)
Viral exanthem rash	6100 (0.9)	0 (0.0)	549 (0.6)	2575 (0.9)	2913 (1.1)	80 (0.3)	0 (0.0)	22 (0.4)	37 (0.3)	21 (0.6)
Dermatologic disorder	13 050 (2.0)	82 (4.0)	1554 (1.7)	5882 (2.0)	5396 (2.0)	799 (3.4)	70 (9.6)	187 (3.0)	372 (2.9)	168 (4.9)
Anemia	50 794 (7.6)	39 (1.9)	9696 (10.7)	30 777 (10.6)	9990 (3.6)	7258 (31.3)	23 (3.2)	1608 (26.0)	4805 (37.7)	804 (23.3)
Vertically transmitted infections	417 (0.1)	47 (2.3)	205 (0.2)	144 (0.0)	19 (0.0)	248 (1.1)	24 (3.3)	165 (2.7)	59 (0.5)	0 (0.0)
Neonatal sepsis	194 (0.0)	145 (7.1)	35 (0.0)	11 (0.0)	3 (0.0)	160 (0.7)	127 (17.4)	19 (0.3)	11 (0.1)	3 (0.1)
Birth-related conditions	221 (0.0)	109 (5.4)	59 (0.1)	44 (0.0)	7 (0.0)	224 (1.0)	27 (3.7)	122 (2.0)	72 (0.6)	2 (0.1)

Multiple diagnoses were possible for each patient, so total percentages may sum up more than 100%.

Abbreviations: LRTI, lower respiratory tract infection; URTI, upper respiratory tract infection.

Focusing exclusively on the original CISM demographic area, the MCBIRs per 1000 residents per year for malaria in outpatients and inpatients were 264.5 (95% CI, 263.0–266.1) and 14.4 (95% CI, 14.0–14.7), respectively; for LRTI, 110.4 (95% CI, 109.4–111.4) and 10.4 (95% CI, 10.1–10.7); and for AGI, 65.6 (95% CI, 64.9–66.4) and 4.8 (95% CI, 4.6–5.0) (Supplementary Data 5).

The diagnoses with highest mortality odds among outpatients were meningoencephalitis (aOR, 18.4; 95% CI, 10.4–30.6; CFR 8.0%), seizures with fever (aOR, 12.1; 95% CI, 8.9–16.1; CFR 2.1%), and malnutrition (aOR, 14.5; 95% CI, 11.9–17.7; CFR 4.5%), as well as sepsis and neonatal sepsis. Among inpatients, the diagnoses most strongly associated with mortality were sepsis (aOR, 7.1; 95% CI, 5.3–9.3; CFR 15.7%), neonatal sepsis (aOR, 6.7; 95% CI, 3.3–13.0; CFR 9.4%), and *P. jirovecii* infection (aOR, 6.7; 95% CI, 3.2–12.9; CFR 14.3%), as well as meningoencephalitis, malnutrition, and HIV infection (Table 4; Supplementary Data 6).

**Table 4.** Logistic Regression Models for 7-Day Mortality Odds of Diagnoses, Stratified by Outpatient Clinic Visits and Inpatients, Adjusted by Age Group, Sex, and Season

Diagnoses	Outpatients			Inpatients		
	aOR	95% CI	*P* Value	aOR	95% CI	*P* Value
Age group	…	…		…	…	…
Late child	Baseline	…		Baseline	…	…
Early child	1.88	1.54–2.31	<.001	0.85	0.64–1.15	.29
Infant	3.66	2.93–4.58	<.001	1.20	0.88–1.64	.25
Neonate	8.11	4.70–13.5	<.001	0.54	0.27–1.02	.066
Sex	…	…		…	…	…
Male	Baseline	…		Baseline	…	…
Female	0.98	0.86–1.12	.82	1	0.84–1.20	>.9
Season	…	…		…	…	…
Dry	Baseline	…		Baseline	…	…
Rainy	1.07	0.93–1.22	.35	0.95	0.79–1.15	.60
Sepsis	10.9	3.12–28.8	<.001	7.05	5.28–9.32	<.001
Nonspecific febrile syndrome	0.42	0.30–0.58	<.001	0.29	0.07–0.78	.037
Tuberculosis	1.92	0.57–4.81	.22	0.96	0.52–1.66	>.9
*Pneumocystis*	6.98	1.69–19.0	.001	6.69	3.17–12.9	<.001
Upper respiratory tract infection	0.32	0.24–0.41	<.001	0.39	0.15–0.81	.023
Lower respiratory tract infection	1.86	1.56–2.20	<.001	1.08	0.87–1.33	.50
Noninfectious respiratory condition	0.60	0.15–1.59	.39	1.98	0.60–4.83	.19
Acute gastrointestinal infection	2.13	1.77–2.56	<.001	1.38	1.08–1.76	.01
Parasitic gastrointestinal infection	0.38	0.15–0.78	.019	0	0.00–0.00	>.9
Malaria	0.84	0.70–1.00	.052	0.57	0.44–0.73	<.001
Meningoencephalitis	18.4	10.4–30.6	<.001	4.48	2.90–6.75	<.001
Seizures with fever	12.1	8.91–16.1	<.001	1.3	0.87–1.90	.18
Ear infections	0.81	0.51–1.22	.34	0.49	0.17–1.08	.12
Ocular infections	0.27	0.11–0.52	<.001	0	0.00–0.00	>.9
HIV infection	4.62	3.30–6.33	<.001	1.62	1.24–2.10	<.001
Malnutrition	14.5	11.9–17.7	<.001	2.36	1.86–2.98	<.001
Viral exanthem rash	0.32	0.05–1.00	.11	1.59	0.38–4.42	.44
Dermatologic disorder	0.95	0.55–1.51	.83	0.05	0.00–0.24	.004
Anemia	2.57	2.16–3.04	<.001	1.03	0.81–1.31	.8
Vertically transmitted infections	3.91	2.22–6.47	<.001	1.2	0.57–2.22	.60
Neonatal sepsis	10.1	4.93–19.80	<.001	6.65	3.28–13.0	<.001
Birth-related conditions	9.08	4.66–16.60	<.001	1.28	0.57–2.50	.51

Excluded from the model due to missing data: 6868 in outpatients (from a total of 664 223), 71 in inpatients (from a total of 23 166).

Abbreviation: aOR, adjusted odds ratio.

Among the most frequent diagnoses, malaria showed CFRs of 0.13% in outpatients and 1.17% in inpatients, with lower odds of mortality compared with children not diagnosed with malaria (outpatients: aOR, 0.8; 95% CI, 0.7–1.0; inpatients: aOR, 0.6; 95% CI, 0.4–0.7). For LRTI, CFRs were 0.41% in outpatients and 2.84% in inpatients. For outpatients, higher odds of mortality were associated with having an LRTI diagnosis (aOR, 1.74; 95% CI, 1.47–2.06). For AGI, CFRs were 0.44% in outpatients and 3.63% in inpatients, with higher odds in both groups (outpatients: aOR, 2.1; 95% CI, 1.8–2.6; inpatients: aOR, 1.4; 95% CI, 1.1–1.8).

## DISCUSSION

This 17-year analysis of surveillance data, integrating clinical and demographic information, provides a thorough description of the epidemiology of febrile illness and its associated mortality among outpatient visits and hospitalized children in a low-income, rural district in Southern Mozambique. Malaria, respiratory tract infections, unspecified fever, and AGI were the most common diagnoses among outpatients and inpatients, contributing to an extensive disease burden that has progressively declined over time. Other conditions, such as sepsis and meningoencephalitis, were less frequent but associated with higher mortality. Severe malnutrition and HIV infection were identified as relevant risk factors for fatal outcomes.

The overall epidemiological trend revealed a decrease in outpatient visits, hospital admissions, and total deaths over time, consistent with previous reports from Manhiça district [25]. This decline was particularly pronounced for malaria, LRTI, and AGI. Manhiça is a paradigm of a resource-limited setting whereby combined research and public health initiatives have significantly influenced population health. Examples include the introduction of the *Haemophilus influenzae* type b (2009), pneumococcal conjugate (2013), and rotavirus (2015) vaccines into the Mozambican immunization program [25], clinical trials with thousands of participants for a malaria candidate vaccine (2003–2004) and intermittent preventive treatment (2002–2005) that contributed to national policies [30–32], research on malaria epidemiology and treatments [33–35], studies on bacterial infections [36–43], the establishment of a cause-of-death surveillance system using postmortem sampling, and elaborating data-to-action strategies to prevent child deaths [25]. In our study, 7-day mortality was 0.1% among outpatients and 2.2% among inpatients, with the highest risk observed in neonates and young infants, which may be explained by their greater vulnerability. These mortality rates are lower than those reported in other studies on febrile illnesses in LMICs, which have an estimated 0.3% mortality in uncomplicated fever [44], 3.5%–7% among inpatients [18, 21, 22, 45–47], and up to 11% in severely ill cases [12, 48].

We found a distribution of diagnoses comparable to other malaria-endemic regions. Among outpatients, the highest burden of disease was due to respiratory infections, malaria, and unspecified fever. In general, respiratory infections account for more than half of fevers in LMICs, but about one-quarter meet WHO criteria for pneumonia, and only 5% are radiologically confirmed [1, 18, 49]. URTIs accounted for 27.8% of outpatient diagnoses and were nearly 3 times more frequent than LRTIs (10.1%). LRTIs were more common in younger children. Among inpatients, LRTIs were diagnosed in nearly one-third of cases (27.5%), which is similar to reports from other sites (18%–37%) [21, 22, 45], depending on the malaria endemicity, as the prevalence of both conditions may covary [15, 50]. Malaria was very frequent in our setting, accounting for >33% of outpatient visits and an MCBIR of ∼250 cases per 1000 residents per year. Depending on the endemicity, other sites reported a prevalence of 3% (Ethiopia) [20], 10% (Tanzania) [18], or >30% (Burkina Faso) [21]. Malaria-related hospitalizations represented a substantial proportion of total admissions (49.0% and MCBIR ∼15 cases per 1000 residents per year). These figures have decreased over time and have been discussed in detail elsewhere [26, 51]. AGI was diagnosed in 6.0% of outpatients and 12.4% of inpatients, consistent with findings from other studies [18, 19, 22, 45, 47, 49]. This suggests that, despite rotavirus vaccination and infection prevention strategies, the disease burden remains high. Rotavirus was estimated to cause 35% and 20% of admitted and outpatient cases of pediatric diarrhea, respectively, in Manhiça district in 2007–2012 [52, 53]. Rotavirus vaccine coverage in Mozambique increased from 17% in 2014 to 58%–80% in 2023, according to the WHO [54]. However, in the Manhiça district, vaccine coverage presented higher estimates, encompassing most children and resulting in a prevalence reduction of 40% for AGI and 84% for rotavirus-confirmed cases [55]. Aside from rotavirus, previous studies in the district highlighted the relevant role of *Cryptosporidium* spp. in moderate to severe disease [52]. Other diseases, such as meningoencephalitis, comprised a small number of cases (<1%–2%), as in other reports from Kenya and Tanzania [1, 18, 47]. Unspecified fever accounted for 15.2% of outpatient diagnoses, which falls within the range reported in other pediatric studies in resource-limited settings (3.0%–25.5%), depending on the extensiveness of laboratory evaluation [18, 45, 49]. Unspecified fever was more frequent in neonates (22.4% of outpatients and 16.6% of inpatients) compared with other age groups, highlighting the challenges of clinical evaluation in this population. Previous reports in Manhiça district reported 8% of bloodstream infections in admitted children <2 years old and 9.9% in admitted young infants <90 days old [9, 42]. However, the sparse diagnosis of urinary tract infections (UTIs) is concerning. Although UTI may account for a limited number of cases [1, 18, 20, 49], it has been well described as a cause of occult bacteremia and fever without source in young infants and is frequently overlooked in LMICs despite being diagnosable with minimal equipment and training [1, 56].

Although common illnesses such as malaria, LRTI, and AGI were associated with lower individual mortality risk than less frequent conditions like sepsis or meningitis, they accounted for the greatest absolute number of deaths, consistent with previous reports in Sub-Saharan Africa [12, 18, 21]. The CFRs among inpatients in our study were 1.2% for malaria, 2.8% for LRTI, 3.6% for AGI, 9.4% for neonatal sepsis, 15.7% for sepsis in older children, and 13.0% for meningoencephalitis, similar to other studies in hospitalized patients [12, 22, 47]. Importantly, malnutrition and HIV infection were present in 10.2% and 7.4% of inpatients, respectively, strongly contributing to inpatient mortality. This is consistent with findings from postmortem studies in LMICs, where malnutrition and HIV were reported as the underlying causes of death in 16.5% and 11.9% of children U5 [57]. According to WHO estimates for 2023, although new infections have declined over time, Mozambique continues to face a severe HIV epidemic including ∼150 000 children with HIV, only 67% of whom receive antiretroviral therapy [58]. In Manhiça district, a ∼39% HIV prevalence in adults was reported [59]. Our study occurred in the era pre–integrase strand transfer inhibitor regimens, as these were not introduced in Mozambique until 2021–2022 [60]. Clinical signs were found to align with the corresponding diagnoses in both prevalence and severity, as reflected in their associated mortality odds. Key predictors of severe disease included the presence of seizures, edema, dehydration, pallor or anemia, and any reduction in level of consciousness. These findings support WHO recommendations on the clinical assessment of pediatric patients [61, 62].

The main strength of this study is its large, long-term cohort of well-characterized patients, with standardized data collection enabling detailed analysis of diagnostic patterns across outpatient and inpatient care, age groups, and time. Integration with the DSS allowed robust mortality assessment and calculation of MCBIRs, rarely reported in LMICs. Although limited to a single district in Southern Mozambique, we believe these findings may be generalizable to similar low-resource settings with comparable malaria endemicity and may inform public health and health system interventions. However, several limitations should be acknowledged. We could not determine the microbiological etiology of most diseases, as the analysis was based on clinical codes and uniformly available complimentary exams were lacking; in particular, information regarding urine cultures was not systematically collected. Restricting to patients with permanent identification numbers may have introduced selection bias with respect to the entire district population, and not all district health care facilities participated in the MSS. The severity of clinical symptoms may be slightly overestimated due to the selection of the later-stage visit if multiple visits occurred in a clinical episode. However, this occurred in <6% of visits. MCBIRs may be underestimated in neonates who were possibly admitted before MSS registration. HIV infection, malnutrition, and sepsis were not systematically coded in outpatient diagnoses, likely leading to underestimation of their true burden. Finally, we lacked data on family education, socioeconomic status, transportation, and traditional medicine use, all factors that could shape health-seeking behavior and outcomes.

In summary, malaria, LRTI, AGI, malnutrition, HIV infection, sepsis, and meningitis were the primary contributors to febrile illness and mortality among pediatric patients in a rural district of Southern Mozambique. Despite the encouraging decline in disease burden, the fact that most of these conditions are preventable [57], or at least treatable and manageable, must not be overlooked. Sustained efforts are needed at multiple levels of health care delivery and public health policy. This analysis underscores the value of continuous morbidity and epidemiological surveillance and highlights the urgent need for strengthened prevention strategies, early diagnostic tools, and improved evidence-based strategies for the clinical management of febrile children.

## Supplementary Material

ofaf724_Supplementary_Data

## Supplementary Data

Supplementary materials are available at *Open Forum Infectious Diseases* online. Consisting of data provided by the authors to benefit the reader, the posted materials are not copyedited and are the sole responsibility of the authors, so questions or comments should be addressed to the corresponding author.
