# Supplementary material for: Epidemiology and Outcomes of Pediatric Fever in a Rural District of Southern Mozambique: 17 Years of Morbidity Surveillance
Source: Open Forum Infect Dis. 2025 Nov 28;12(12):ofaf724. doi: 10.1093/ofid/ofaf724 (PMC12696371; doi:10.1093/ofid/ofaf724)
Supplement: ofaf724_Supplementary_Data [file ofaf724_supplementary_data.docx]

# SUPPLEMENTARY MATERIAL

**Article title**: Epidemiology and outcomes of pediatric fever in a rural district of Southern Mozambique: 17 years of morbidity surveillance

**Open Forum Infectious Diseases**

## **Supplementary Material 1**. Additional information on methods.

Details on malaria diagnosis definition

For outpatients, malaria diagnosis was defined as the presence of fever (measured temperature at triage ≥37.5 ºC, history of current, or referred fever) and a positive test for malaria, this is, the presence of parasitemia either by semi-quantitative thick smear observation or parasite density quantification. A positive rapid diagnostic test was also considered if thick smear and parasite density variables were missing. For inpatients, malaria diagnosis was defined as a clinical diagnosis of malaria coded in ICD-10 in any of the four diagnostic variables, and a positive test for malaria as defined for outpatients.

Calculation of mortality proportions, case fatality ratios (CFR), and minimum community-based incidence rates (MCBIR)

Mortality proportions by year were calculated, for outpatients, as the number of deaths within seven days of the outpatient visit in a specific year divided by the number of clinical episodes in that year; and for inpatients, as the number of deaths in hospitalized divided by the number of hospital admissions. Case fatality ratios (CFRs) were calculated as the number of patients who died within seven days of the outpatient visit having a specific diagnosis divided by the total number of patients with known outcome and the same diagnosis, in total population for outpatients, and in admitted for inpatients. Absconders and transfers were not excluded for mortality proportions by year and CFR calculations.

Minimum community-based incidence rates (MCBIRs) included only patients resident in the original CISM DSS catchment area (pre-expansion) for compatibility between years.^1^ Thus, MCBIRs include only a subset of the study populations, whereas all other results include all patients. MCBIRs for different diagnoses were calculated as the yearly number of patients with a specific diagnosis resident in the original area, divided by the cumulative individual time at risk for year inferred from the DSS, as done for other studies in this cohort.^2,3^ MCBIR denominators were constructed using census-derived exposure intervals, split at each seasonal change and at individual age group transitions. Patients did not contribute to calculations when they were outside the study area. After the time defined as a clinical episode (seven days), any subsequent visit or admission was considered unrelated to a previous one and modelled as a new index visit/admission.

Ethical approvals

This analysis was approved by CISM’s Internal Scientific Committee (ref. CCI/030/MAI/2020). The MDSS was approved by the Manhiça local administrative authorities, the Institutional Ethics Review Board (ref. CIBS_CISM/01/12), and the National Committee for Bioethics in Health of Mozambique (ref. 174/CNBS/12). All residents (or their legal guardians) of the study area provided signed individual informed consent to be enrolled in the MDSS.

Organization and grouping of ICD-10 diagnostic codes according to authors’ criteria

Note that only clinically relevant diagnosis or those with more than 0.1% cases were reported in the results section. *Final malaria diagnosis required further testing as described above.

| **ICD-10 code** | **Name** | **Group name** |
| --- | --- | --- |
| A39 | Meningococcal infection | Sepsis |
| A40 | Streptococcal sepsis |  |
| A41 | Other sepsis |  |
| B37.7 | Candidal sepsis |  |
| R57 | Shock, not elsewhere classified |  |
| R57.2 | Septic shock |  |
| R57.9 | Shock, unspecified |  |
| R65 | Systemic Inflammatory Response Syndrome |  |
| A68 | Relapsing fevers | Non-specific febrile syndrome |
| R50 | Fever of other and unknown origin |  |
| R50.0 | Fever with chills |  |
| R50.1 | Persistent fever |  |
| R50.8 | Other specified fever |  |
| R50.9 | Fever, unspecified |  |
| A15 | Respiratory tuberculosis, bacteriologically and histologically confirmed | Tuberculosis |
| A16 | Respiratory tuberculosis, not confirmed bacteriologically or histologically |  |
| A31.0 | Pulmonary mycobacterial infection |  |
| B59 | Pneumocystosis (J17.3*) | Pneumocystis |
| J17.3 | Pneumonia in parasitic diseases |  |
| J00 | Acute nasopharyngitis [common cold] | Upper respiratory tract infection |
| J04 | Acute laryngitis and tracheitis |  |
| J05 | Acute obstructive laryngitis [croup] and epiglottitis |  |
| J06 | Acute upper respiratory infections of multiple and unspecified sites |  |
| J09 | Influenza due to certain identified influenza virus |  |
| J10 | Influenza due to other identified influenza virus |  |
| J11 | Influenza, virus not identified |  |
| J01 | Acute sinusitis |  |
| J02 | Acute pharyngitis |  |
| J03 | Acute tonsillitis |  |
| J36 | Peritonsillar abscess |  |
| B00.2 | Herpesviral gingivostomatitis and pharyngotonsillitis |  |
| J12 | Viral pneumonia, not elsewhere classified | Lower respiratory tract infection and pneumonia |
| J13 | Pneumonia due to *Streptococcus pneumoniae* |  |
| J14 | Pneumonia due to *Haemophilus influenzae* |  |
| J15 | Bacterial pneumonia, not elsewhere classified |  |
| J16 | Pneumonia due to other infectious organisms, not elsewhere classified |  |
| J17 | Pneumonia in diseases classified elsewhere |  |
| J18 | Pneumonia, organism unspecified |  |
| J20 | Acute bronchitis |  |
| J21 | Acute bronchiolitis |  |
| J22 | Unspecified acute lower respiratory infection |  |
| J40 | Bronchitis, not specified as acute or chronic |  |
| J85 | Abscess of lung and mediastinum |  |
| J86 | Pyothorax |  |
| J90 | Pleural effusion, not elsewhere classified |  |
| R06.0 | Dyspnoea |  |
| R06.2 | Wheezing |  |
| J45 | Asthma | Non-infectious respiratory condition |
| J46 | Status asthmaticus |  |
| J47 | Bronchiectasis |  |
| J68 | Respiratory conditions due to inhalation of chemicals, gases, fumes and vapours |  |
| J69 | Pneumonitis due to solids and liquids |  |
| A36 | Diphtheria | Other respiratory infections |
| A37 | Whooping cough |  |
| B37.1 | Pulmonary candidiasis |  |
| B38.0 | Acute pulmonary coccidioidomycosis |  |
| B38.2 | Pulmonary coccidioidomycosis, unspecified |  |
| B39 | Histoplasmosis |  |
| B40 | Blastomycosis |  |
| B41 | Paracoccidioidomycosis |  |
| B44 | Aspergillosis |  |
| B45.0 | Pulmonary cryptococcosis |  |
| B46.0 | Pulmonary mucormycosis |  |
| A00 | Cholera | Acute gastrointestinal infection |
| A01 | Typhoid and paratyphoid fevers |  |
| A02 | Other Salmonella infections |  |
| A03 | Shigellosis |  |
| A04 | Other bacterial intestinal infections |  |
| A05 | Other bacterial foodborne intoxications, not elsewhere classified |  |
| A08 | Viral and other specified intestinal infections |  |
| A08.0 | Rotaviral enteritis |  |
| A09 | Other gastroenteritis and colitis of infectious and unspecified origin |  |
| A06 | Amoebiasis | Parasitic gastrointestinal infection |
| A07 | Other protozoal intestinal diseases |  |
| B77 | Ascariasis |  |
| B65 | Schistosomiasis [bilharziasis] |  |
| B67 | Echinococcosis |  |
| B76 | Hookworm diseases |  |
| B78 | Strongyloidiasis |  |
| B79 | Trichuriasis |  |
| B80 | Enterobiasis |  |
| B81 | Other intestinal helminthiases, not elsewhere classified |  |
| B82 | Unspecified intestinal parasitism |  |
| B83.9 | Helminthiasis, unspecified |  |
| B15 | Acute hepatitis A | Hepatitis and hepatobiliary disorders |
| B16 | Acute hepatitis B |  |
| B17 | Other acute viral hepatitis |  |
| K81 | Cholecystitis |  |
| K85 | Acute pancreatitis |  |
| K35 | Acute appendicitis | Appendicitis and acute abdomen |
| K36 | Other appendicitis |  |
| K37 | Unspecified appendicitis |  |
| K65 | Peritonitis |  |
| R10.0 | Acute abdomen |  |
| B50 | Plasmodium falciparum malaria | Malaria* |
| B50.0 | Plasmodium falciparum malaria with cerebral complications |  |
| B50.8 | Other severe and complicated Plasmodium falciparum malaria |  |
| B50.9 | Plasmodium falciparum malaria, unspecified |  |
| B51 | Plasmodium vivax malaria |  |
| B52 | Plasmodium malariae malaria |  |
| B53 | Other parasitologically confirmed malaria |  |
| B54 | Unspecified malaria |  |
| A39.0 | Meningococcal meningitis | Meningoencephalitis |
| G00 | Bacterial meningitis, not elsewhere classified |  |
| G00.0 | Haemophilus meningitis |  |
| G00.1 | Pneumococcal meningitis |  |
| G00.2 | Streptococcal meningitis |  |
| G00.3 | Staphylococcal meningitis |  |
| G00.8 | Other bacterial meningitis |  |
| G00.9 | Bacterial meningitis, unspecified |  |
| G01 | Meningitis in bacterial diseases classified elsewhere |  |
| G03.9 | Meningitis, unspecified |  |
| A17 | Tuberculosis of nervous system |  |
| A84 | Tick-borne viral encephalitis |  |
| A85 | Other viral encephalitis, not elsewhere classified |  |
| A86 | Unspecified viral encephalitis |  |
| A87 | Viral meningitis |  |
| A89 | Unspecified viral infection of central nervous system |  |
| A99 | Unspecified viral haemorrhagic fever |  |
| B01.1 | Varicella meningitis |  |
| B01.1 | Varicella encephalitis |  |
| B02.0 | Zoster encephalitis |  |
| B02.1 | Zoster meningitis |  |
| B02.2 | Zoster with other nervous system involvement |  |
| B05.0 | Measles complicated by encephalitis |  |
| B05.1 | Measles complicated by meningitis |  |
| B45 | Cryptococcosis |  |
| B45.1 | Cerebral cryptococcosis |  |
| B58.2 | Toxoplasma meningoencephalitis |  |
| G02 | Meningitis in other infectious and parasitic diseases classified elsewhere |  |
| G02.1 | Meningitis in mycoses |  |
| G03 | Meningitis due to other and unspecified causes |  |
| G03.0 | Nonpyogenic meningitis |  |
| G04 | Encephalitis, myelitis and encephalomyelitis |  |
| G04.0 | Acute disseminated encephalitis |  |
| G04.2 | Bacterial meningoencephalitis and meningomyelitis, not elsewhere classified |  |
| G04.9 | Encephalitis, myelitis and encephalomyelitis, unspecified |  |
| G05 | Encephalitis, myelitis and encephalomyelitis in diseases classified elsewhere |  |
| G05.2 | Encephalitis, myelitis and encephalomyelitis in other infectious and parasitic diseases classified elsewhere |  |
| G06 | Intracranial and intraspinal abscess and granuloma |  |
| G08 | Intracranial and intraspinal phlebitis and thrombophlebitis |  |
| R29.1 | Meningismus |  |
| R56 | Convulsions, not elsewhere classified | Seizures with fever |
| R56.0 | Febrile convulsions |  |
| R56.8 | Other and unspecified convulsions |  |
| F44 | Dissociative [conversion] disorders | Other neurological disorders |
| G26 | Extrapyramidal and movement disorders in diseases classified elsewhere |  |
| G40 | Epilepsy |  |
| G40.0 | Localization-related (focal)(partial) idiopathic epilepsy and epileptic syndromes with seizures of localized onset |  |
| G40.1 | Localization-related (focal)(partial) symptomatic epilepsy and epileptic syndromes with simple partial seizures |  |
| G40.3 | Generalized idiopathic epilepsy and epileptic syndromes |  |
| G40.4 | Other generalized epilepsy and epileptic syndromes |  |
| G40.9 | Epilepsy, unspecified |  |
| G41 | Status epilepticus |  |
| G41.9 | Status epilepticus, unspecified |  |
| R40 | Somnolence, stupor and coma |  |
| H60 | Otitis externa | Ear infections |
| H65 | Nonsuppurative otitis media |  |
| H66 | Suppurative and unspecified otitis media |  |
| H67 | Otitis media in diseases classified elsewhere |  |
| H70 | Mastoiditis and related conditions |  |
| B30 | Viral conjunctivitis | Ocular infections |
| H01.0 | Blepharitis |  |
| H04.0 | Dacryoadenitis |  |
| H05.0 | Acute inflammation of orbit |  |
| H10 | Conjunctivitis |  |
| H19.1 | Herpesviral keratitis and keratoconjunctivitis (B00.5+) |  |
| B20 | Human immunodeficiency virus [HIV] disease resulting in infectious and parasitic diseases | HIV infection |
| B21 | Human immunodeficiency virus [HIV] disease resulting in malignant neoplasms |  |
| B22 | Human immunodeficiency virus [HIV] disease resulting in other specified diseases |  |
| B23 | Human immunodeficiency virus [HIV] disease resulting in other conditions |  |
| B24 | Unspecified human immunodeficiency virus [HIV] disease |  |
| R75 | Laboratory evidence of human immunodeficiency virus [HIV] |  |
| Z21 | Asymptomatic human immunodeficiency virus [HIV] infection status |  |
| E40 | Kwashiorkor | Malnutrition |
| E41 | Nutritional marasmus |  |
| E42 | Marasmic kwashiorkor |  |
| E43 | Unspecified severe protein-energy malnutrition |  |
| E44 | Protein-energy malnutrition of moderate and mild degree |  |
| E45 | Retarded development following protein-energy malnutrition |  |
| E46 | Unspecified protein-energy malnutrition |  |
| E50 | Vitamin A deficiency | Nutritional deficiencies |
| E52 | Niacin deficiency [pellagra] |  |
| E53 | Deficiency of other B group vitamins |  |
| E54 | Ascorbic acid deficiency |  |
| E55 | Vitamin D deficiency |  |
| E56 | Other vitamin deficiencies |  |
| E58 | Dietary calcium deficiency |  |
| E59 | Dietary selenium deficiency |  |
| E60 | Dietary zinc deficiency |  |
| E61 | Deficiency of other nutrient elements |  |
| E90 | Nutritional and metabolic disorders in diseases classified elsewhere |  |
| A18 | Tuberculosis of other organs | Other mycobacterial infections |
| A19 | Miliary tuberculosis |  |
| A31.8 | Other mycobacterial infections |  |
| A31.9 | Mycobacterial infection, unspecified |  |
| A90 | Dengue fever [classical dengue] | Viral hemorrhagic fevers |
| A91 | Dengue haemorrhagic fever |  |
| A92 | Other mosquito-borne viral fevers |  |
| A95 | Yellow fever |  |
| A96 | Arenaviral haemorrhagic fever |  |
| A98 | Other viral haemorrhagic fevers, not elsewhere classified |  |
| A99 | Unspecified viral haemorrhagic fever |  |
| B01 | Varicella [chickenpox] | Viral exanthem rash |
| B05 | Measles |  |
| B06 | Rubella [German measles] |  |
| B25 | Cytomegaloviral disease |  |
| B26 | Mumps |  |
| B27 | Infectious mononucleosis |  |
| R30.0 | Dysuria | Urinary infection |
| R30.9 | Painful micturition, unspecified |  |
| A51 | Early syphilis | Sexually-transmitted diseases |
| A52 | Late syphilis |  |
| A53 | Other and unspecified syphilis |  |
| A54 | Gonococcal infection |  |
| A55 | Chlamydial lymphogranuloma (venereum) |  |
| A56 | Other sexually transmitted chlamydial diseases |  |
| A58 | Granuloma inguinale |  |
| A59 | Trichomoniasis |  |
| A60 | Anogenital herpesviral [herpes simplex] infection |  |
| A63 | Other predominantly sexually transmitted diseases, not elsewhere classified |  |
| A64 | Unspecified sexually transmitted disease |  |
| A82 | Rabies | Other specific infections |
| A23 | Brucellosis |  |
| A27 | Leptospirosis |  |
| A28 | Other zoonotic bacterial diseases, not elsewhere classified |  |
| A32 | Listeriosis |  |
| A38 | Scarlet fever |  |
| A42 | Actinomycosis |  |
| A43 | Nocardiosis |  |
| A44 | Bartonellosis |  |
| A46 | Erysipelas |  |
| A69 | Other spirochaetal infections |  |
| A71 | Trachoma |  |
| A74 | Other diseases caused by chlamydiae |  |
| A75 | Typhus fever |  |
| A77 | Spotted fever [tick-borne rickettsioses] |  |
| A78 | Q fever |  |
| A80 | Acute poliomyelitis |  |
| B55 | Leishmaniasis |  |
| B56 | African trypanosomiasis |  |
| B58 | Toxoplasmosis |  |
| B68 | Taeniasis |  |
| B69 | Cysticercosis |  |
| B72 | Dracunculiasis |  |
| B73 | Onchocerciasis |  |
| B74 | Filariasis |  |
| B75 | Trichinellosis |  |
| I38 | Endocarditis, valve unspecified |  |
| I40 | Acute myocarditis |  |
| I41 | Myocarditis in diseases classified elsewhere |  |
| M00 | Pyogenic arthritis |  |
| M01 | Direct infections of joint in infectious and parasitic diseases classified elsewhere |  |
| M13 | Other arthritis |  |
| M60 | Myositis |  |
| M86 | Osteomyelitis |  |
| A31.1 | Cutaneous mycobacterial infection | Dermatologic disorder |
| B02 | Zoster [herpes zoster] |  |
| B07 | Viral warts |  |
| B08 | Other viral infections characterized by skin and mucous membrane lesions, not elsewhere classified |  |
| B35 | Dermatophytosis |  |
| B36 | Other superficial mycoses |  |
| B37.2 | Candidiasis of skin and nail |  |
| B37.9 | Candidiasis, unspecified |  |
| B42 | Sporotrichosis |  |
| B47 | Mycetoma |  |
| B85 | Pediculosis and phthiriasis |  |
| B86 | Scabies |  |
| B87 | Myiasis |  |
| L00 | Staphylococcal scalded skin syndrome |  |
| L01 | Impetigo |  |
| L02 | Cutaneous abscess, furuncle and carbuncle |  |
| L03 | Cellulitis |  |
| L04 | Acute lymphadenitis |  |
| M79.3 | Panniculitis, unspecified |  |
| D50 | Iron deficiency anaemia | Anemia |
| D51 | Vitamin B12 deficiency anaemia |  |
| D52 | Folate deficiency anaemia |  |
| D55 | Anaemia due to enzyme disorders |  |
| D56 | Thalassaemia |  |
| D57 | Sickle-cell disorders |  |
| D58 | Other hereditary haemolytic anaemias |  |
| D59 | Acquired haemolytic anaemia |  |
| D62 | Acute posthaemorrhagic anaemia |  |
| D63 | Anaemia in chronic diseases classified elsewhere |  |
| D64 | Other anaemias |  |
| R23.1 | Pallor |  |
| D69 | Purpura and other haemorrhagic conditions | Other hematologic disorder |
| D80 | Immunodeficiency with predominantly antibody defects |  |
| D81 | Combined immunodeficiencies |  |
| D82 | Immunodeficiency associated with other major defects |  |
| D83 | Common variable immunodeficiency |  |
| D84 | Other immunodeficiencies |  |
| C80 | Malignant neoplasm without specification of site | Oncologic disorder |
| C46 | Kaposi's sarcoma |  |
| C95 | Leukaemia of unspecified cell type |  |
| C94 | Other leukaemias of specified cell type |  |
| C96 | Other and unspecified malignant neoplasms of lymphoid, haematopoietic and related tissue |  |
| D48 | Neoplasm of uncertain or unknown behaviour of other and unspecified sites |  |
| E05 | Thyrotoxicosis [hyperthyroidism] | Endocrine disorder |
| E06 | Thyroiditis |  |
| E10 | Type 1 diabetes mellitus |  |
| E11 | Type 2 diabetes mellitus |  |
| E12 | Malnutrition-related diabetes mellitus |  |
| E13 | Other specified diabetes mellitus |  |
| E14 | Unspecified diabetes mellitus |  |
| E24 | Cushing's syndrome |  |
| E25 | Adrenogenital disorders |  |
| E30 | Disorders of puberty, not elsewhere classified |  |
| E34 | Other endocrine disorders |  |
| E35 | Disorders of endocrine glands in diseases classified elsewhere |  |
| E66 | Obesity |  |
| A33 | Tetanus neonatorum | Tetanus |
| A35 | Other tetanus |  |
| A50 | Congenital syphilis | Vertically-transmitted infections |
| P00.2 | Fetus and newborn affected by maternal infectious and parasitic diseases |  |
| P37 | Other congenital infectious and parasitic diseases |  |
| P35 | Congenital viral diseases |  |
| P36 | Bacterial sepsis of newborn | Neonatal sepsis |
| P23 | Congenital pneumonia | Respiratory disorders of the newborn |
| P22 | Respiratory distress of newborn |  |
| P24 | Neonatal aspiration syndromes |  |
| P26 | Pulmonary haemorrhage originating in the perinatal period |  |
| R09.2 | Respiratory arrest |  |
| P28.3 | Primary sleep apnoea of newborn |  |
| P28.4 | Other apnoea of newborn |  |
| P28.5 | Respiratory failure of newborn |  |
| P77 | Necrotizing enterocolitis of fetus and newborn | Digestive disorders of the newborn |
| P92 | Feeding problems of newborn |  |
| P90 | Convulsions of newborn | Neonatal seizures |
| P38 | Omphalitis of newborn with or without mild haemorrhage | Other neonatal infections |
| P39 | Other infections specific to the perinatal period |  |
| P00 | Fetus and newborn affected by maternal conditions that may be unrelated to present pregnancy | Birth-related conditions |
| P01 | Fetus and newborn affected by maternal complications of pregnancy |  |
| P02 | Fetus and newborn affected by complications of placenta, cord and membranes |  |
| P03 | Fetus and newborn affected by other complications of labour and delivery |  |
| P04 | Fetus and newborn affected by noxious influences transmitted via placenta or breast milk |  |
| P20 | Intrauterine hypoxia |  |
| P21 | Birth asphyxia |  |

## **Supplementary Material 2**. Annual 7-day mortality proportion over time (2004-2020) for visits to outpatient clinic and inpatients (table and figure).

*Legend: Line graph plotting blue lines that corresponds to outpatient clinic visits, and red lines, to the inpatients. The solid lines represent the mortality proportion per year (in percentage, y-axis); the dotted lines represent the total number of outpatient visits or hospital admissions (x-axis).*

*Alt text: Line graph showing the annual 7-day mortality proportion (solid lines, left y-axis in percentage) and the total number of patients (dotted lines, right y-axis) for inpatients (red lines) and outpatient clinic visits (blue lines) over 2004 to 2020. Total outpatient visits show a large fluctuation, with a high of 52,159 in 2012 and a low of 22,605 in 2020. The mortality proportion decreased from 0.29% in 2004 to 0.1% in 2020. For inpatients, the total number of patients decreased from 2,273 in 2004 to 435 in 2020, and mortality fluctuated between 1.2% to 2.9%.*


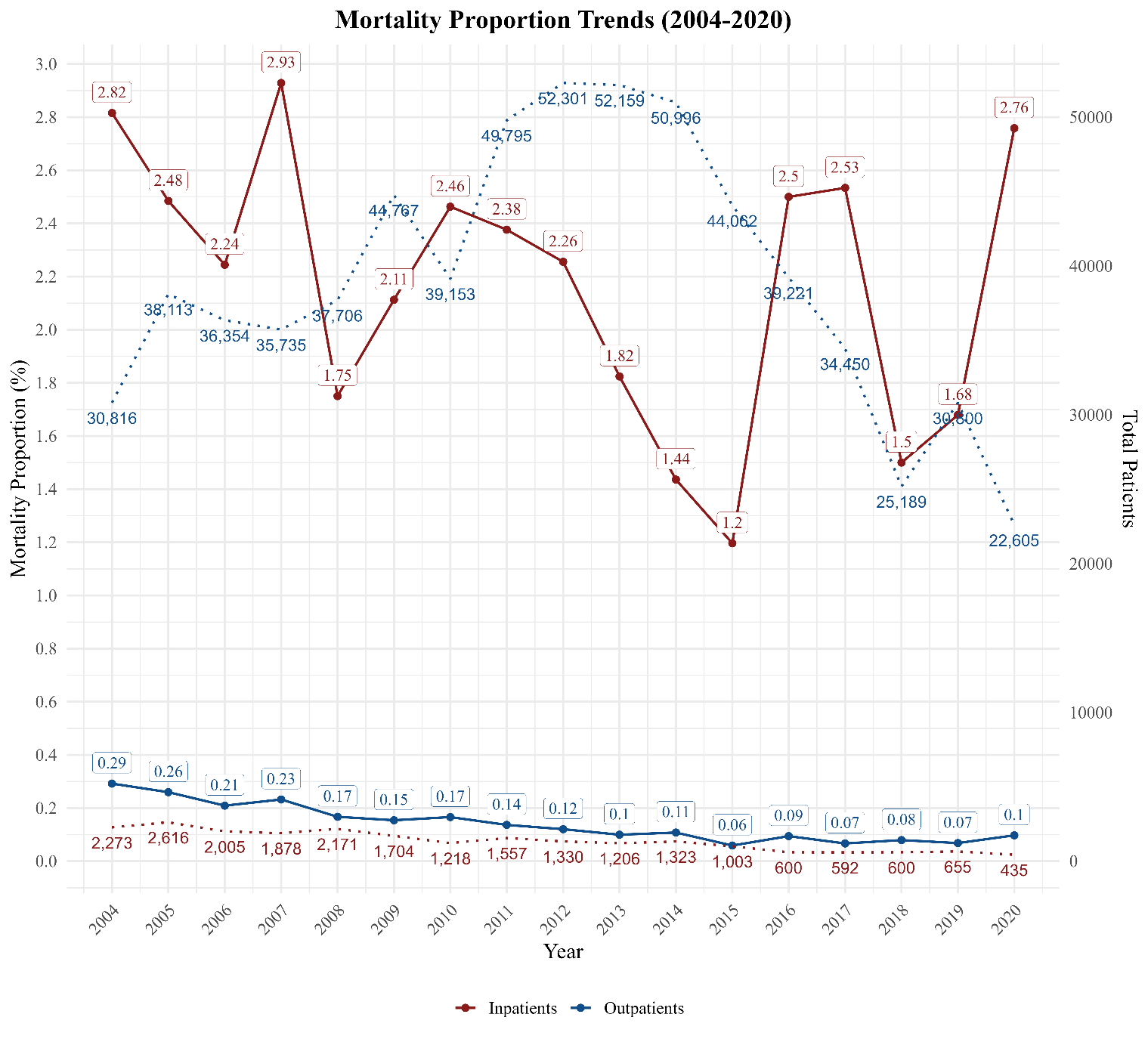


|  | **OUTPATIENTS** | | | | **INPATIENTS** | | | |
| --- | --- | --- | --- | --- | --- | --- | --- | --- |
| **Year** | **Deaths** | **Total visits** | **Visits per 1,000 children <15 years** | **Mortality proportion (%)** | **Deaths** | **Total admissions** | **Admissions per 1,000 children <15 years** | **Mortality proportion (%)** |
| 2004 | 90 | 30816 | 9883 | 0.29 | 64 | 2273 | 729 | 2.82 |
| 2005 | 99 | 38113 | 9600 | 0.26 | 65 | 2616 | 659 | 2.48 |
| 2006 | 76 | 36354 | 8460 | 0.21 | 45 | 2005 | 467 | 2.24 |
| 2007 | 83 | 35735 | 7705 | 0.23 | 55 | 1878 | 405 | 2.93 |
| 2008 | 63 | 37706 | 7421 | 0.17 | 38 | 2171 | 427 | 1.75 |
| 2009 | 69 | 44767 | 8631 | 0.15 | 36 | 1704 | 329 | 2.11 |
| 2010 | 65 | 39153 | 6920 | 0.17 | 30 | 1218 | 215 | 2.46 |
| 2011 | 68 | 49795 | 8715 | 0.14 | 37 | 1557 | 272 | 2.38 |
| 2012 | 63 | 52301 | 9470 | 0.12 | 30 | 1330 | 241 | 2.26 |
| 2013 | 52 | 52159 | 10415 | 0.10 | 22 | 1206 | 241 | 1.82 |
| 2014 | 55 | 50996 | 8895 | 0.11 | 19 | 1323 | 231 | 1.44 |
| 2015 | 26 | 44062 | 6081 | 0.06 | 12 | 1003 | 138 | 1.20 |
| 2016 | 37 | 39221 | 4805 | 0.09 | 15 | 600 | 74 | 2.50 |
| 2017 | 24 | 34451 | 3565 | 0.07 | 15 | 592 | 61 | 2.53 |
| 2018 | 20 | 25189 | 2373 | 0.08 | 9 | 600 | 57 | 1.50 |
| 2019 | 21 | 30800 | 2969 | 0.07 | 11 | 655 | 63 | 1.68 |
| 2020 | 22 | 22605 | 2933 | 0.10 | 12 | 435 | 56 | 2.76 |

## **Supplementary Material 3**. Logistic regression models for 7-day mortality odds of clinical signs, stratified by outpatient clinic visits and inpatients, adjusted by age group, sex, and season.

*Legend: Excluded from the model due to missing data: 21,274 in outpatients (from a total of 664,223*)*, 2,076 in inpatients (from a total of 23,166*)*. aOR: adjusted Odds Ratio; CI: Confidence interval.*

| **SYMPTOMS** | **OUTPATIENTS** | | | **INPATIENTS** | | |
| --- | --- | --- | --- | --- | --- | --- |
|  | **aOR** | **95% CI** | **p-value** | **aOR** | **95% CI** | **p-value** |
| **Age group** |  |  |  |  |  |  |
| Late childhood | Baseline |  |  | Baseline |  |  |
| Early childhood | 1.58 | 1.26-1.99 | <0.001 | 0.66 | 0.48-0.92 | 0.011 |
| Infant | 3.66 | 2.87-4.68 | <0.001 | 1.12 | 0.80-1.58 | 0.53 |
| Neonate | 19.10 | 12.0-29.40 | <0.001 | 1.56 | 0.84-2.76 | 0.14 |
| **Sex** |  |  |  |  |  |  |
| Male | Baseline |  |  | Baseline |  |  |
| Female | 0.99 | 0.85-1.14 | 0.85 | 1.00 | 0.81-1.22 | >0.9 |
| **Season** |  |  |  |  |  |  |
| Dry | Baseline |  |  | Baseline |  |  |
| Rainy | 1.03 | 0.88-1.20 | 0.73 | 0.96 | 0.78-1.18 | 0.70 |
| **Axillary temperature ≥37.5º** | 1.52 | 1.30-1.77 | <0.001 | 0.88 | 0.72-1.09 | 0.25 |
| **Cough** | 0.77 | 0.65-0.91 | 0.002 | 0.65 | 0.51-0.84 | <0.001 |
| **Difficulty breathing** | 3.67 | 2.74-4.88 | <0.001 | 2.90 | 2.07-4.04 | <0.001 |
| **Tachypnea** | 1.79 | 1.50-2.14 | <0.001 | 1.09 | 0.85-1.40 | 0.50 |
| **Chest indrawing** | 2.27 | 1.69-3.07 | <0.001 | 1.55 | 1.12-2.17 | 0.01 |
| **Diarrhea** | 1.68 | 1.37-2.06 | <0.001 | 1.64 | 1.25-2.14 | <0.001 |
| **Vomiting** | 1.53 | 1.26-1.85 | <0.001 | 1.22 | 0.96-1.55 | 0.11 |
| **Decreased skin turgor** | 0.90 | 0.57-1.43 | 0.67 | 1.40 | 0.91-2.14 | 0.12 |
| **Moderate/Severe dehydration** | 4.06 | 2.64-6.11 | <0.001 | 1.07 | 0.67-1.67 | 0.80 |
| **Seizures** | 5.95 | 4.47-7.83 | <0.001 | 1.76 | 1.30-2.36 | <0.001 |
| **Neck stiffness** | 2.41 | 0.92-5.50 | 0.052 | 2.38 | 1.15-4.49 | 0.012 |
| **Lethargy** | 4.52 | 3.53-5.77 | <0.001 | Not included | | |
| **Prostration** | Not included | | | 4.15 | 3.27-5.25 | <0.001 |
| **Stopped drinking or breastfeeding** | Not included | | | 1.66 | 1.28-2.13 | <0.001 |
| **Pallor** | 2.79 | 2.26-3.42 | <0.001 | 1.36 | 1.06-1.74 | 0.013 |
| **Jaundice** | 1.26 | 0.52-2.69 | 0.58 | 1.15 | 0.56-2.16 | 0.70 |
| **Edema** | 5.84 | 4.21-7.94 | <0.001 | 3.15 | 2.15-4.51 | <0.001 |
| **Severe underweight** | 3.30 | 2.73-3.97 | <0.001 | 2.62 | 2.07-3.31 | <0.001 |

## **Supplementary Material 4**. Heatmap of co-occurrence of diagnoses (overlapping) in outpatient clinic visits*.*

*Legend: Percentages are calculated using each diagnosis as the denominator for each row (y-axis) or columns (x-axis). Interpretation example: "HIV infection" (y-axis) and "Tuberculosis" (x-axis): Percentage = (Number of patients with both) / (Number with " HIV infection ")*100; "Tuberculosis" (y-axis) and "HIV infection" (x-axis): Percentage = (Number of patients with both) / (Number with "Tuberculosis")*100.*

*Alt text: Heatmap showing the percentage of co-occurrence between 23 diagnoses among outpatient clinic visits. Diagnoses are displayed along both the X and Y axes using descriptive labels such as 'Sepsis,' 'HIV infection,' and 'Tuberculosis.' Each cell indicates the percentage of patients with the diagnosis listed on the Y-axis who also have the diagnosis on the X-axis. Darker green shades represent higher percentages, while lighter shades indicate lower percentages, with numeric values inside each tile. The main diagonal shows 100%, reflecting patients with the same diagnosis in both axes. Off-diagonal cells display asymmetric percentages depending on the selected denominator.*


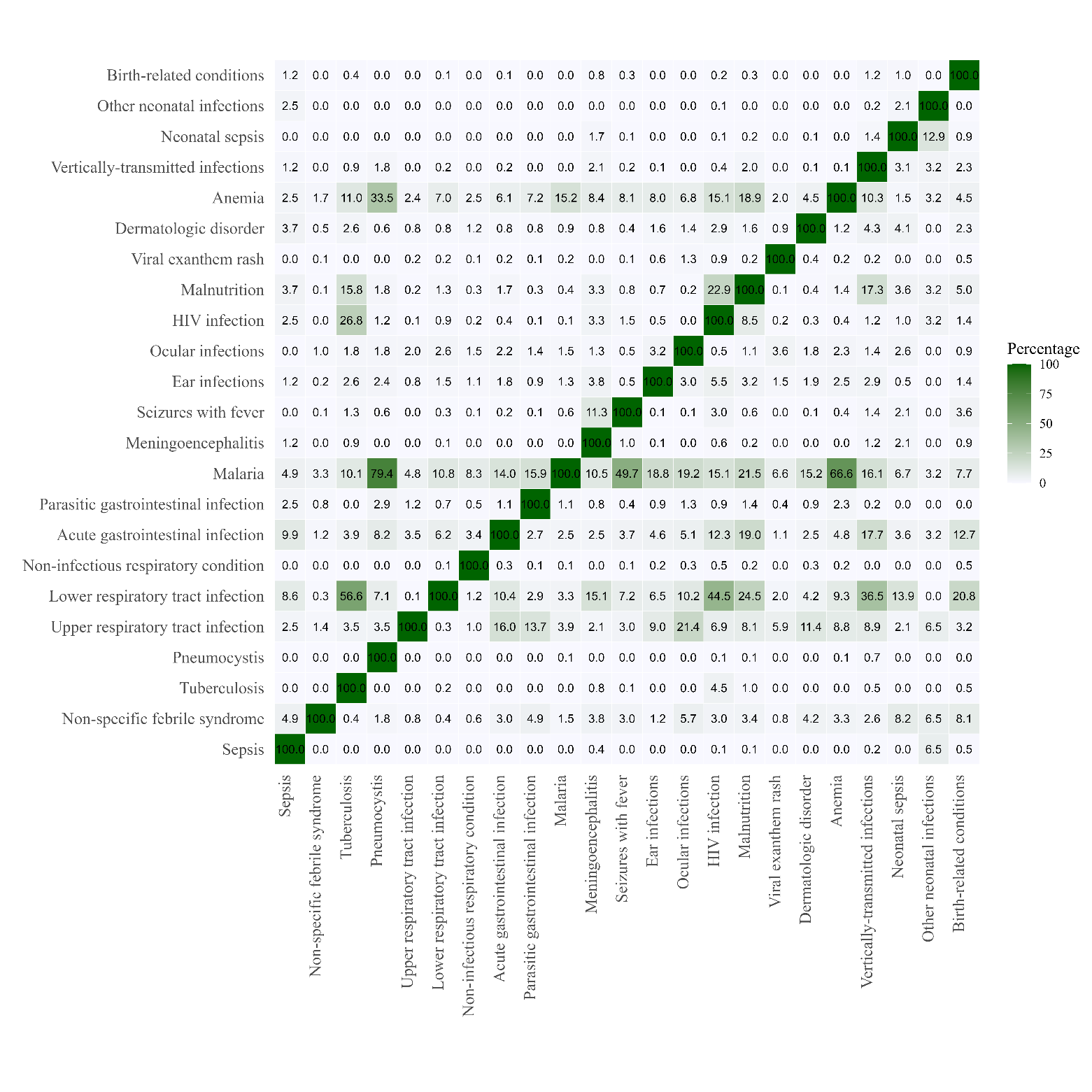


## **Supplementary Material 5**. Minimum community-based incidence rates (MCBIRs) for selected diagnoses in outpatient clinic visits and inpatients resident at CISM original study area.

*Legend: MCBIRs are shown per 1,000 residents with 95% confidence intervals. Abbreviations: LRTI: Lower respiratory tract infection. URTI: Upper respiratory tract infection. NA: not available.*

|  | **OUTPATIENTS** | | | | | **INPATIENTS** | | | | |
| --- | --- | --- | --- | --- | --- | --- | --- | --- | --- | --- |
|  | **Total** | **Neonate** | **Infant** | **Early childhood** | **Late childhood** | **Total** | **Neonate** | **Infant** | **Early childhood** | **Late childhood** |
| **Non-specific febrile syndrome** | 132.5 (131.4-133.5) | 80.2 (73.0-88.2) | 163.7 (160.4-167.0) | 154.1 (152.4-156.0) | 102.1 (100.7-103.6) | 0.9 (0.8-1.0) | 19.8 (16.4-24.0) | 1.8 (1.5-2.2) | 0.8 (0.7-0.9) | 0.2 (0.1-0.2) |
| **URTI** | 285.4 (283.8-287.0) | 96.5 (88.5-105.2) | 439.4 (434.0-444.8) | 351.5 (348.7-354.2) | 174.8 (172.9-176.7) | 1.0 (1.0-1.2) | NA | NA | NA | NA |
| **LRTI and pneumonia** | 110.4 (109.4-111.4) | 41.5 (36.4-47.4) | 300.6 (296.2-305.1) | 142.2 (140.5-143.9) | 18.8 (18.2-19.5) | 10.4 (10.1-10.7) | 14.0 (11.1-17.5) | 35.8 (34.3-37.3) | 11.4 (11.0-11.9) | 1.1 (0.9-1.2) |
| **Acute gastrointestinal infection** | 65.6 (64.9-66.4) | 16.6 (13.5-20.5) | 198.7 (195.2-202.4) | 81.8 (80.5-83.1) | 7.6 (7.3-8.1) | 4.8 (4.6-5.0) | 4.2 (2.7-6.3) | 18.8 (17.7-19.9) | 4.8 (4.5-5.1) | 0.2 (0.1-0.2) |
| **Malaria** | 264.5 (263.0-266.1) | 19.3 (15.8-23.4) | 193.8 (190.3-197.4) | 317.5 (314.9-320.1) | 241.6 (239.3-243.9) | 14.4 (14.0-14.7) | 4.9 (3.3-7.2) | 27.0 (25.7-28.3) | 21.0 (20.4-21.7) | 3.9 (3.6-4.2) |
| **Meningoencephalitis** | 0.3 (0.3-0.4) | 1.7 (0.9-3.3) | 1.0 (0.8-1.3) | 0.3 (0.2-0.3) | 0.1 (0.1-0.2) | 0.3 (0.3-0.4) | 3.0 (1.8-4.9) | 1.1 (0.8-1.4) | 0.2 (0.1-0.3) | 0.1 (0.04-0.1) |

## **Supplementary Material 6**. Case Fatality Ratios (CFRs) for diagnoses in outpatient clinic visits and inpatients. Fatality includes death within seven days of the outpatient clinic visit.

*Legend: LRTI: Lower respiratory tract infection. URTI: Upper respiratory tract infection.*

|  | **OUTPATIENTS** | | | **INPATIENTS** | | |
| --- | --- | --- | --- | --- | --- | --- |
|  | **Deaths** | **Diagnoses** | **CFR (%)** | **Deaths** | **Diagnoses** | **CFR (%)** |
| **Sepsis** | 4 | 81 | 4.94 | 76 | 483 | 15.73 |
| **Non-specific febrile syndrome** | 47 | 100,707 | 0.05 | 3 | 548 | 0.55 |
| **Tuberculosis** | 4 | 228 | 1.75 | 14 | 304 | 4.61 |
| **Pneumocystis** | 3 | 170 | 1.76 | 11 | 77 | 14.29 |
| **URTI** | 73 | 184,926 | 0.04 | 6 | 747 | 0.80 |
| **LRTI and pneumonia** | 275 | 67,191 | 0.41 | 181 | 6,379 | 2.84 |
| **Non-infectious respiratory condition** | 3 | 3,527 | 0.09 | 4 | 110 | 3.64 |
| **Acute gastrointestinal infection** | 178 | 40,173 | 0.44 | 104 | 2,862 | 3.63 |
| **Parasitic gastrointestinal infection** | 6 | 15,943 | 0.04 | 0 | 128 | 0.00 |
| **Malaria** | 286 | 222,764 | 0.13 | 133 | 11,350 | 1.17 |
| **Meningoencephalitis** | 19 | 239 | 7.95 | 33 | 254 | 12.99 |
| **Seizures with fever** | 56 | 2,682 | 2.09 | 30 | 1,544 | 1.94 |
| **Ear infections** | 22 | 15,827 | 0.14 | 5 | 383 | 1.31 |
| **Ocular infections** | 7 | 17,102 | 0.04 | 0 | 126 | 0.00 |
| **HIV infection** | 51 | 1,353 | 3.77 | 96 | 1,723 | 5.57 |
| **Malnutrition** | 162 | 3,635 | 4.46 | 128 | 2,362 | 5.42 |
| **Viral exanthem rash** | 2 | 6,100 | 0.03 | 3 | 80 | 3.75 |
| **Dermatologic disorder** | 16 | 13,050 | 0.12 | 1 | 799 | 0.13 |
| **Anemia** | 202 | 50,794 | 0.40 | 112 | 7,258 | 1.54 |
| **Vertically-transmitted infections** | 17 | 417 | 4.08 | 10 | 248 | 4.03 |
| **Neonatal sepsis** | 14 | 194 | 7.22 | 15 | 160 | 9.38 |
| **Birth-related conditions** | 14 | 221 | 6.33 | 8 | 224 | 3.57 |

# REFERENCES

1. Nhacolo A, Jamisse E, Augusto O, et al. Cohort Profile Update: Manhica Health and Demographic Surveillance System (HDSS) of the Manhica Health Research Centre (CISM). *Int J Epidemiol.* May 17 2021;50(2):395.

2. Guinovart C, Sigaúque B, Bassat Q, et al. The epidemiology of severe malaria at Manhiça District Hospital, Mozambique: a retrospective analysis of 20 years of malaria admissions surveillance data. *The Lancet Global Health.* 2022;10(6):e873-e881.

3. Balanza N, Hunguana A, Ajanovic S, et al. Paediatric healthcare in Manhiça district through a gender lens: a retrospective analysis of 17 years of morbidity and demographic surveillance data. *J Glob Health.* Feb 21 2025;15:04010.
